# DNA metabarcoding uncovers fungal diversity in soils of protected and non-protected areas on Deception Island, Antarctica

**DOI:** 10.1038/s41598-020-78934-7

**Published:** 2020-12-15

**Authors:** Luiz Henrique Rosa, Thamar Holanda da Silva, Mayara Baptistucci Ogaki, Otávio Henrique Bezerra Pinto, Michael Stech, Peter Convey, Micheline Carvalho-Silva, Carlos Augusto Rosa, Paulo E. A. S. Câmara

**Affiliations:** 1 Laboratório de Microbiologia Polar & Conexões Tropicais, Departamento de Microbiologia, Instituto de Ciências Biológicas, Universidade Federal de Minas Gerais, P. O. Box 486, Belo Horizonte, MG CEP 31270-901 Brazil; 2 Departamento de Biologia Celular, Universidade de Brasília, Brasília, Brazil; 3 Naturalis Biodiversity Center, Leiden, The Netherlands; 4 British Antarctic Survey, NERC, High Cross, Madingley Road, Cambridge, CB3 0ET UK; 5 Departamento de Botânica, Universidade de Brasília, Brasília, Brazil

**Keywords:** Environmental sciences, Environmental impact, Environmental microbiology, Soil microbiology

## Abstract

We assessed soil fungal diversity at two sites on Deception Island, South Shetland Islands, Antarctica using DNA metabarcoding analysis. The first site was a relatively undisturbed area, and the second was much more heavily impacted by research and tourism. We detected 346 fungal amplicon sequence variants dominated by the phyla *Ascomycota*, *Basidiomycota*, *Mortierellomycota* and *Chytridiomycota*. We also detected taxa belonging to the rare phyla *Mucoromycota* and *Rozellomycota*, which have been difficult to detect in Antarctica by traditional isolation methods. *Cladosporium* sp., *Pseudogymnoascus roseus*, *Leotiomycetes* sp. 2, *Penicillium* sp., *Mortierella* sp. 1, *Mortierella* sp. 2, *Pseudogymnoascus appendiculatus* and *Pseudogymnoascus* sp. were the most dominant fungi. In addition, 440,153 of the total of 1,214,875 reads detected could be classified only at the level of Fungi. In both sampling areas the DNA of opportunistic, phytopathogenic and symbiotic fungi were detected, which might have been introduced by human activities, transported by birds or wind, and/or represent resident fungi not previously reported from Antarctica. Further long-term studies are required to elucidate how biological colonization in the island may be affected by climatic changes and/or other anthropogenic influences.

## Introduction

The pristine environments of Antarctica are used as field laboratories to support taxonomic, ecological, evolutionary and biotechnological studies. Antarctic environments experience multiple extreme conditions including low temperatures, acidic and alkaline pH, ultra-oligotrophic conditions, freeze–thaw cycles, salinity stress, desiccation, wind abrasion and high radiation levels^1^ and, for these reasons, offer unique opportunities to study the diversity of fungi^2^.

In the latter part of the Twentieth Century, the Antarctic Peninsula region was one of the regions of the planet most affected by climatic changes. Deception Island, located in the South Shetland Islands is one of very few active volcanoes in the Antarctic Treaty area. Two summer-only research stations are presently active on the island (the Argentinian Decepción and Spanish Gabriel de Castilla). In addition, a shore-based whaling station operated in Whalers Bay in the early Twentieth Century^3^. The combination of unique geology, history, biota and aesthetic values, as well as the active presence of multiple national operators, underlie the designation of the entire island as an Antarctic Specially Managed Area (ASMA 4). In addition, Deception Island includes two Antarctic Specially Protected Areas (ASPAs), designated as ASPAs 140 (terrestrial, formed of multiple sub-sites) and 145 (marine). Deception Island is one of the best-known locations in Antarctica, visited by both researchers and tourists^4^, with more than 55,489 tourists visiting the island in the summer of 2018–2019 (https://iaato.org/tourism-statistics-327mnsyd), which generates pressure on its ecosystems. The island is an exceptional location even within Antarctica, as it is a young volcanic island formed less than 100 kya^5^ and still in the process of biological colonization.

The majority of mycological studies in Antarctica to date have focused on cultivable species, mainly represented by taxa of the phylum *Ascomycota* and its anamorphs, followed by *Basidiomycota*, *Mortierellomycota*, *Mucoromycota*, *Chytridiomycota* and *Glomeromycota*^2^. In Antarctica, different fungal assemblages contribute to complex ecological networks, including saprophytic, mutualistic and parasitic taxa, all of which are able to survive under various extreme environmental conditions^6,7^. However, despite the recognized importance of fungal diversity in Antarctica, few studies have applied metabarcoding approaches using high throughput sequencing (HTS). The present study aimed to characterize and compare fungal diversity assessed using metabarcoding in soil at two sites on Deception Island, (1) a relatively undisturbed site within the terrestrial Antarctic Specially Protected Area (ASPA) 140 and (2) a disturbed site in Whalers Bay subject to considerable visitor pressure and hence greater human impact.

## Methods

### Soil sampling

**Figure 1:**
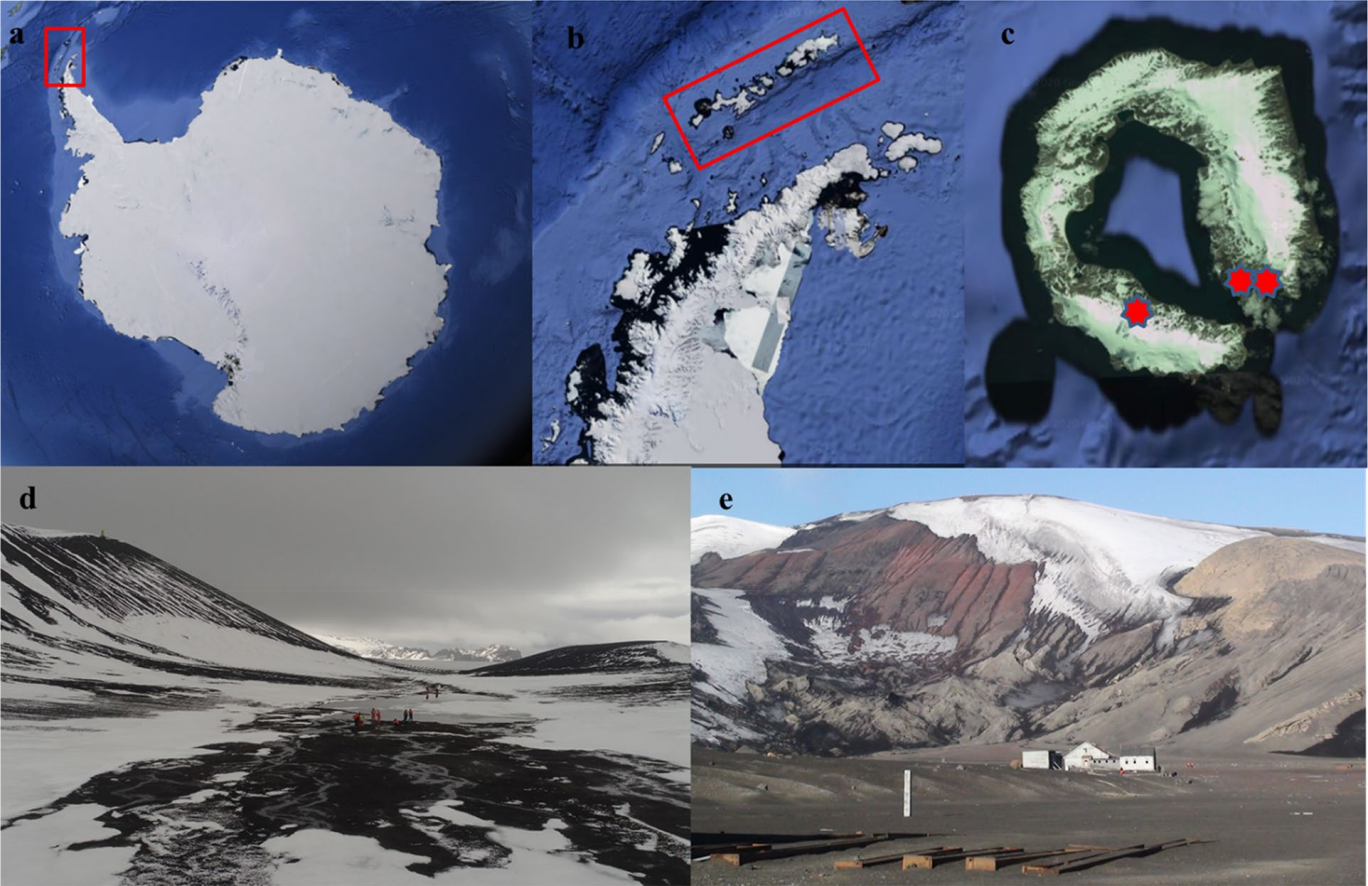
Satellite images (**a**–**c**) (obtained in Google Earth Pro, 2019) and sites were the soil where sampled. (**a**) Antarctic Continent with the northern Antarctic Peninsula inside the red rectangle, (**b**) Antarctic Peninsula with the South Shetland Islands archipelago inside the red rectangle, (**c**) Deception Island with the sites *ASPA 140 and **Whalers Bay, (**d**) Antarctic Specially Protected Area 140 subsite B (protected area close to Crater Lake—62° 06′ 08.6′′ S; 57° 55′10.4′′ W), and (**e**) Whalers Bay (non-protected area, WB—62° 58′ 52.0′′ S; 60° 39′ 52.9′′ W). Photos (**d**,**e**) by L.H. Rosa.

Soil samples were collected from two sites on Deception Island, South Shetland Islands (Fig. 1). The first was within an Antarctic Specially Protected Area (ASPA) close to Crater Lake [ASPA 140, subsite B], which has relatively low impact from researchers and is not accessible for tourism. The second site was in Whalers Bay, which includes the area of the historical whaling station and former UK research station on the island, and is formally declared a Historic Monument. It is one of the most popular visitor sites in Antarctica for both tourists and national operator personnel. The distance between the two sites is approximately 5 km. Superficial soil samples (approximately 5 cm depth and ca 250 g each) were collected using sterile spatulas and immediately placed in sterilized WhirlPak bags (Sigma-Aldrich, USA) kept at − 20 °C until processing. Seven (non-composite) samples from each site (obtained a minimum of 10 m from each other) were collected for use in DNA studies, totaling 14 samples in total.

### DNA extraction and analysis, and fungal identification

Total DNA was extracted from environmental samples using the QIAGEN Power Soil Kit, following the manufacturer’s instructions. Extracted DNA was used as template for generating PCR-amplicons. The internal transcribed spacer 2 (ITS2) of the nuclear ribosomal DNA was used as a DNA barcode for molecular species identification^8,9^. PCR-amplicons were generated using the universal primers ITS3 and ITS4^10^ and were sequenced by high-throughput sequencing at Macrogen Inc. (South Korea) on an Illumina MiSeq sequencer, using the MiSeq Reagent Kit v3 (600-cycle) following the manufacturer’s protocol.

Raw fastq files were filtered using BBDuk version 38.34 (BBMap—Bushnell B.—sourceforge.net/projects/bbmap/) to remove Illumina adapters, known Illumina artifacts, and PhiX Control v3 Library. Quality read filtering was carried out using Sickle version 1.33 -q 30-l 50^11^, to trim 3′ or 5′ ends with low Phred quality score, and sequences smaller than 50 bp were also discarded. Remaining sequences were imported to QIIME2 version 2019.10 (https://qiime2.org/) for bioinformatics analyses^12^. The qiime2-dada2 plugin is a complete pipeline that was used to filter, dereplicate, turn paired-end fastq files into merged, and remove chimeras^13^. Taxonomic assignments were determined for amplicon sequence variants (ASVs) using qiime2-feature-classifier^14^ classify-sklearn against the UNITE fungal ITS database version 7.2^15^ and trained with Naive Bayes classifier and a confidence threshold of 98.5%.

Many factors, including extraction, PCR and primer bias, can affect the number of reads^16^, and thus lead to misinterpretation of abundance^17^. However, Giner et al.^18^ concluded that such biases did not affect the proportionality between reads and cell abundance, implying that more reads are linked with higher abundance^19,20^. Therefore, for comparative purposes we used the number of reads as a proxy for relative abundance.

### Fungal diversity and distribution

To quantify species diversity, richness and dominance, we used the following indices: (1) Fisher’s α, (2) Margalef’s and (3) Simpson’s, respectively. The numbers of reads of each amplicon sequence variant (ASV) were used to quantify the fungal taxa present in the soils sampled, where fungal ASVs > 6000 were considered dominant and ≤ 1000 minor components (rare) within the fungal community. Species accumulation curves were assessed using the Mao Tao index. All diversity index calculations were performed using PAST, version 1.90^21^. Results were obtained with 95% confidence, and bootstrap values were calculated from 1000 iterations. Venn diagrams were prepared according to Bardou et al.^22^ to illustrate the comparison of fungal assemblages present in the two sampling areas.

## Results

### Fungal taxonomy

**Figure 2:**
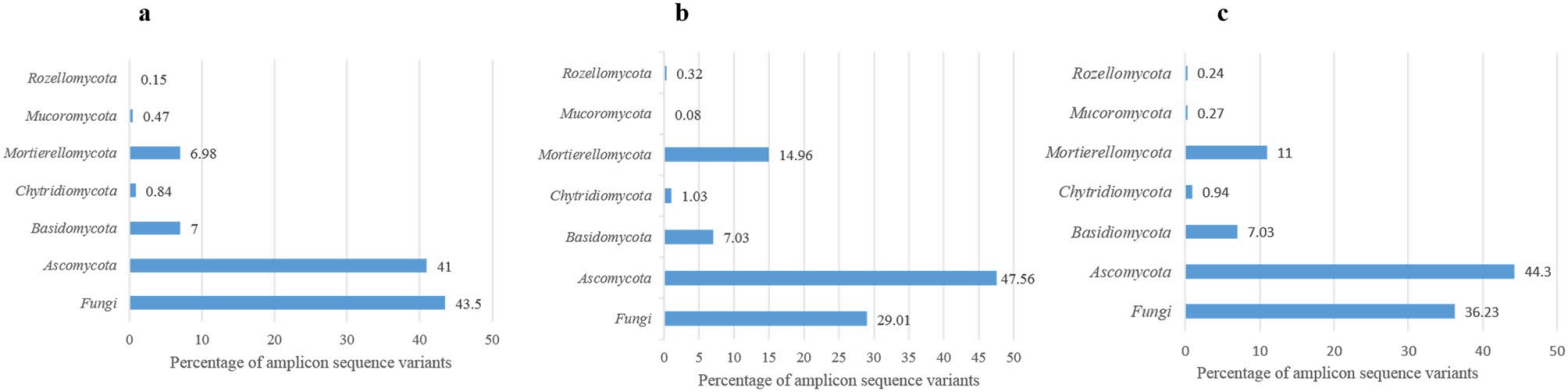
Percentage of fungal amplicon sequence variants (ASVs) at phylum level identified from soil of Deception Island, South Shetland Islands. (**a**) Fungal assemblage of soil in ASPA 140 (non-impacted site), (**b**) fungal assemblage of soil in Whalers Bay (impacted site), and (**c**) total soil fungal community of both sites.

We detected 346 soil fungal amplicon sequence variants (ASVs) in the samples from the two sites on Deception Island (Suppl. Table 1). *Ascomycota*, *Basidiomycota*, *Mortierellomycota* and *Chytridiomycota* dominated the fungal assemblages of both sites at phylum level (Fig. 2). We also detected representatives of the generally rare phyla *Mucoromycota* and *Rozellomycota*, which occurred at moderate dominance in both sites. The ASVs identified as *Cladosporium* sp., *Pseudogymnoascus roseus*, *Leotiomycetes* sp. 2, *Penicillium* sp., *Mortierella* sp. 1, *Mortierella* sp. 2, *Pseudogymnoascus appendiculatus* and *Pseudogymnoascus* sp. were most dominant at genus/species level (with > 30,000 reads). A further 65 ASVs were moderately dominant (> 1000 reads). Twenty-three fungal ASVs could be assigned to only higher hierarchical levels (phylum, class, order or family) when compared with known DNA sequences deposited in the UNITE DNA database^15^ and might represent taxa above the species level new to science and new records for Antarctica. In addition, 440,153 of the total of 1,214,875 reads detected (262,844 in the ASPA and 177,309 in Whalers Bay) could only be classified at the level of Fungi.

### Fungal diversity

**Figure 3:**
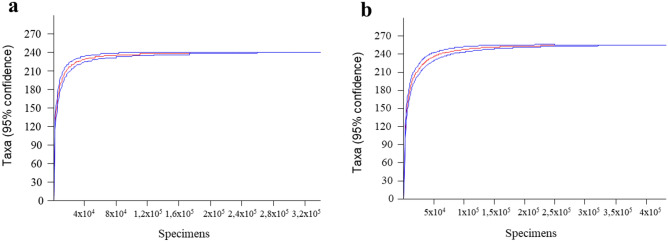
Rarefaction curves for samples from (**a**) ASPA 140 sampling site and (**b**) Whalers Bay site (impacted area) on Deception Island, South Shetland Islands. Blue lines represent confidence limits.

The Mao Tao rarefaction curves reached asymptote for both fungal assemblages (Fig. 3), indicating that the data provided a good description of the diversity present. The fungal assemblages of both sites displayed high diversity, richness, and dominance indices (Table 1) when compared with studies of cultivable fungi present in Antarctic soils^23,24^. That of Whalers Bay had the highest values of each index.

**Table 1:** Diversity indices of fungal assemblages present in soils of the Antarctic Specially Protected Area (ASPA) 140 and Whalers Bay sampling sites on Deception Island, as indicated by numbers of amplicon sequence variants (ASVs) and compared with diversity results of cultivable fungi present in soils of Antarctica.

Indices	ASPA 140^b^	Whalers Bay	Gonçalves et al.^23^^c^	Gomes et al.^24^^c^
Numbers of ASVs^a^	240	255	15^d^	34^d^
Fisher α	25.23	26.25	10.26	4.45
Margalef	18.76	19.57	3.97	3.61
Simpson	0.91	0.93	0.85	0.95

^a^ASVs = amplicon sequence variants.

^b^ASPA = Antarctic Specially Protected Area.

^c^Gonçalves et al.^23^ and Gomes et al.^24^ represent diversity results of cultivable fungi.

^d^Number of fungal taxa detected.

**Figure 4:**
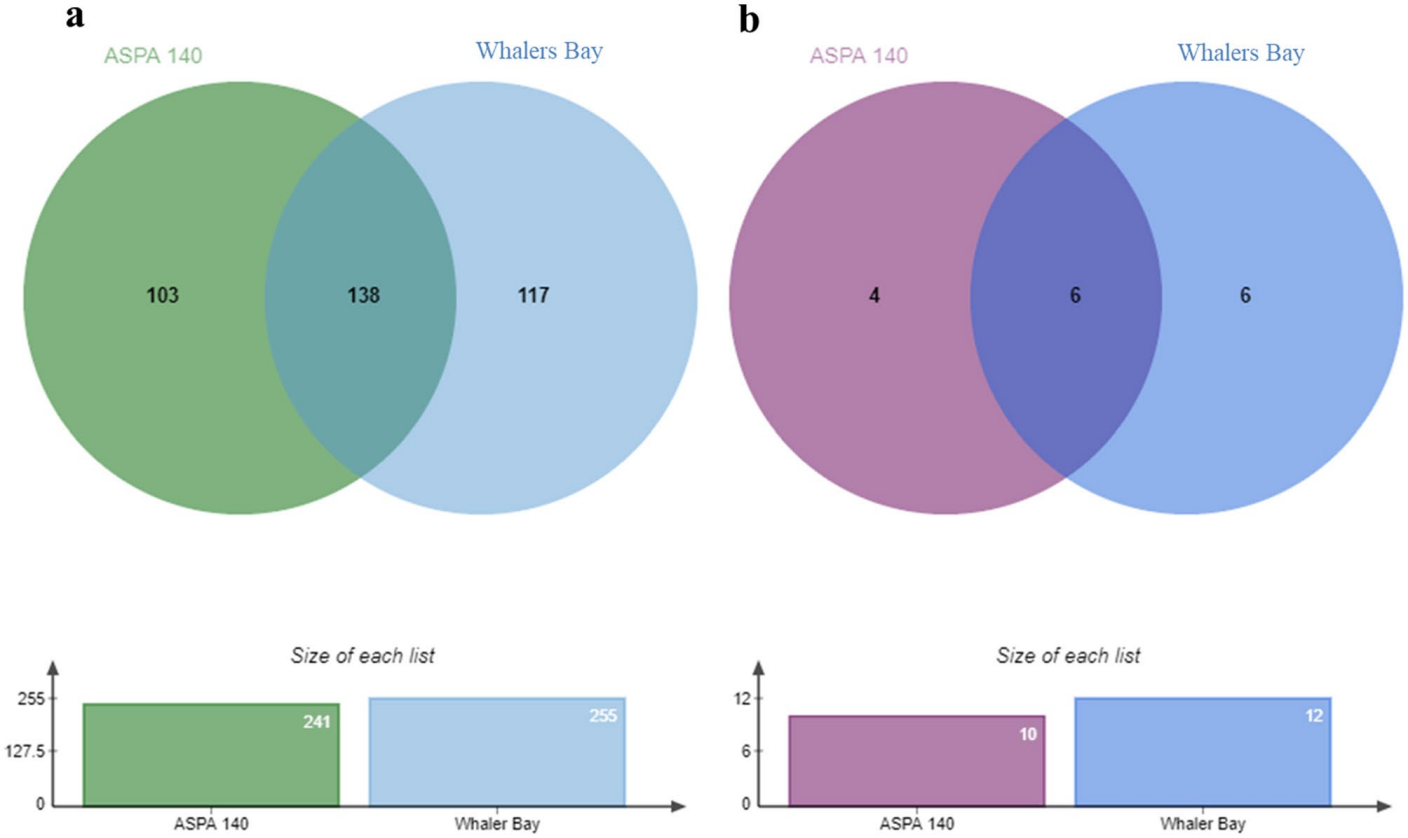
(**a**) Venn diagram showing the total and (**b**) dominant (those with > 6000 reads) fungal taxa distribution between ASPA 140 (non-impacted) and Whalers Bay (impacted) sampling areas.

Of the fungal ASVs characterized, 103 were present only in ASPA 140, 117 in Whalers Bay, with 138 common to both (Fig. 4a), indicating that a small majority of the diversity at both sites was shared between them. The ecological assemblage profiles of exclusive or shared fungi between the two sites did not display significant differences. In both sites the DNA of both cosmopolitan and Antarctic endemic fungi was detected (Suppl. Table 1).

When the dominant fungi (> 6000 reads) were compared between the two sites (Fig. 4b), *Malassezia restricta*, *Mortierella fimbricystis* and *M. antarctica* occurred only in the ASPA samples, and *Leucosporidiella creatinivora*, *Cleistothelebolus nipigonensis*, *Thelebolus globosus*, *Colletotrichum* sp. 1 and *Leotiomycetes* sp. 2 only in the Whalers Bay samples. *Pseudogymnoascus roseus*, *P. appendiculatus*, *Pseudogymnoascus* sp., *Cladosporium* sp., *Mortierella* sp. 2 and *Penicillium* sp. were present in both areas.

The patterns of occurrence of rare taxa (those detected with reads ≤ 1000) in both sites indicated similarities in their ecological status between the assemblages, with the presence of human and animal opportunistic and plant pathogenic taxa (Table 2). In the heavily human impacted Whalers Bay a proportion of the identifiable fungi detected have previously been reported as being opportunistically associated with humans and animals (16 taxa) or phytopathogenic (16). In the soil of ASPA 140, 13 human and animal opportunistic and 12 phytopathogenic taxa were detected. *Aspergillus sydowii*, *Curvularia lunata*, *Malassezia dermatis*, *M. globosa*, *M. restricta*, *M. sympodialis*, *Rhodotorula mucilaginosa* and *Trichosporon asahii* (human and animal associated), and *Aspergillus niger*, *Colletotrichum annellatum*, *Curvularia lunata*, *Gibberella tricincta*, *G. zeae*, *Herpotrichia juniper*, *Nigrospora oryzae*, *Thanatephorus cucumeris* and *Cleistothelebolus nipigonensis* (phytopathogenic) were detected in both sites. We also detected the presence of DNA of 11 lichenized fungi, of which five (*Lecidea cancriformis*, *Psoroma tenue*, *Trimmatothelopsis smaragdula*, *Verrucaria alpicola* and *V. margacea*) occurred in both sites.

**Table 2:** Ecological status of the uncultured fungi recovered from different soil samples of Deception Island, Antarctic Peninsula.

Ecological status	Site/number of reads	
	ASPA 140^a^	Whalers Bay
**Opportunistic human and animal pathogens**		
*Aphanoascus keratinophilus*	0	19
***Aspergillus sydowii***	**427**	**112**
*Aspergillus terreus*	160	0
*Blastobotrys proliferans*	0	12
*Candida parapsilosis*	0	60
***Curvularia lunata***	**91**	**29**
*Cutaneotrichosporon smithiae*	0	64
*Cyphellophora pluriseptata*	24	0
*Exophiala cancerae*	43	0
*Magnusiomyces capitatus*	0	4
***Malassezia dermatis***	**36**	**70**
***Malassezia globosa***	**5831**	**689**
***Malassezia restricta***	**11,413**	**3719**
*Malassezia slooffiae*	35	0
***Malassezia sympodialis***	**364**	**171**
*Malassezia yamatoensis*	0	65
*Papiliotrema laurentii*	0	358
*Pseudallescheria boydii*	21	0
*Pyrenochaeta keratinophila*	0	8
***Rhodotorula mucilaginosa***	**2565**	**2663**
*Sporothrix brasiliensis*	0	46
***Trichosporon asahii***	**158**	**68**
**Plant pathogens**		
***Aspergillus niger***	**292**	**18**
***Colletotrichum annellatum***	**802**	**127**
*Colletotrichum brevisporum*	0	16
*Colletotrichum cliviae*	0	2524
***Curvularia lunata***	**91**	**29**
*Fusarium asiaticum*	0	43
*Fusarium oxysporum*	0	139
*Fusarium solani*	0	115
*Gibberella intricans*	14	0
***Gibberella tricincta***	**4**	**42**
***Gibberella zeae***	**32**	**278**
***Herpotrichia juniper***	**598**	**1074**
*Mycosphaerella tassiana*	0	54
***Nigrospora oryzae***	**2**	**5**
*Peniophora albobadia*	5	0
*Pestalotiopsis trachicarpicola*	0	12
*Pyrenochaeta keratinophila*	0	8
***Thanatephorus cucumeris***	23	182
*Volutella consors*	26	0
***Cleistothelebolus nipigonensis***	980	12,637
**Fungi able to form lichen thalli**		
***Lecidea cancriformis***	129	21
*Lecidea* sp.	0	19
*Parmelina* sp.	64	0
*Placopsis* sp.	35	0
*Psoroma hypnorum*	24	0
***Psoroma tenue***	590	205
***Trimmatothelopsis smaragdula***	73	177
***Verrucaria alpicola***	2305	985
*Verrucaria humida*	0	24
***Verrucaria margacea***	17	30
*Verrucaria nodosa*	253	0

In bold taxa detected in soil of both sites.

^a^ASPA = Antarctic Specially Protected Areas.

## Discussion

### Fungal taxonomy and diversity

In Antarctica, around 1000 fungal species have been described through studies of the macro- and/or micromorphology of colonies and fruiting bodies, and DNA sequencing of mycelia of cultivable fungi^25^. However, according to Amann et al.^26^ and Rappe and Giovannoni^27^, just 0.01–1% of the microbial life present in a given habitat can be characterized using cultivation methods. Magnuson and Lasure^28^ suggested that a rather lower proportion (70–90%) of soil fungi cannot be obtained using culturing methods. Blackwell^29^ and Taylor et al*.*^30^ estimated that, including fungi detected by their environmental DNA, the Kingdom Fungi might include between 5.1 and 6 million species worldwide, respectively.

The majority of mycological studies carried out to date on Deception Island have focused on cultivable fungal diversity. Gonçalves et al.^31^ reported seven fungal taxa present in freshwater in Crater Lake, Held and Blanchette^32^ reported 68 taxa on historic wooden structures in Whalers Bay, Figueredo et al.^4^ identified 17 taxa from soil samples from Fumarole Bay and de Menezes et al.^33^ reported 14 taxa from snow. Baeza et al*.*^34^ used culture-independent techniques to characterize fungal diversity in soils from various different sites in Antarctica, including some samples obtained from the same locations on Deception Island as studied here. They reported 33 taxa, many identified only to genus level, a much lower total than the 346 distinct taxa detected here. Only 10 genera were reported in both studies (*Candida*, *Exophila*, *Herpotrichia*, *Lecidea*, *Malassezia*, *Merozyma*, *Pseudogymnoascus*, *Psoroma*, *Thelebolus* and *Verrucaria*). Baeza et al*.*^32^ reported the most abundant taxa to be *Verticillium* sp., *Xanthophyllomyces dendrorhous*, *Malassezia restricta* and *Circinaria fruticulosa*, differing from the dominant taxa detected in our study (*Cladosporium* sp., *P. roseus*, *Leotiomycetes* sp., *Penicillium* sp., *Mortierella* sp. 1, *Mortierella* sp. 2, *P. appendiculatus* and *Pseudogymnoascus* sp.). Our study differs from that of Baeza et al.^34^ in sample size, techniques used, and PCR bias. Despite these differences, our data confirm the presence of a much higher fungal diversity than reported in previous studies. The observation that many of ASVs could only be classified to higher taxonomic levels, with a significant proportion only to the Kingdom Fungi, suggests that it is likely that Antarctica hosts many as yet unrecognised fungal taxa.

Using number of reads as a proxy measure of abundance, *Ascomycota* was the dominant phylum detected, followed by *Basidiomycota*, *Mortierellomycota* and *Chytridiomycota*. Previous studies of fungal diversity in Antarctic soil have demonstrated the same overall pattern of dominant fungal phyla detected here^6,7,24,35,36^. However, we also detected the presence of taxa from the phyla *Mucoromycota* and *Rozellomycota*, which are not commonly reported in Antarctic soils. Although these phyla have global distributions they are poorly known from Antarctica, when compared with *Ascomycota*, *Basidomycota* and *Mortierellomycota*, and are generally regarded as rare^2^.

Members of the genera *Cladosporium*, *Penicillium* and *Mortierella* dominated the assemblages detected in this study. *Cladosporium* and *Penicillium* include cosmopolitan species detected in Antarctica. *Cladosporium* is one of the largest genera of dematiaceous hyphomycetes^37^, with global distribution. It includes species with many different characteristics, including saprophytic and phytopathogenic taxa^38^. In Antarctica, *Cladosporium* are often associated with the availability of organic matter, such as in moss carpets^39,40^ and the native flowering plant *Colobanthus quitensis* (Kunth.) Bartl. (Caryophyllaceae)^41^. They are broadly distributed in Antarctica, indicating versatility in adaptation to the extreme conditions of the continent, and have been reported from soil, snow, ice, seawater and marine sediments, freshwater and lake sediments, plants and animals^2^.

*Pseudogymnoascus* (syn. *Geomyces*) have been often described from cold habitats of Arctic, alpine, temperate and Antarctic regions^2,42–44^. In Antarctica, *Pseudogymnoascus* is widely distributed and has been reported from both terrestrial and marine ecosystems, including soils^24,42,45^, mosses^39,40,46^, as an endophyte of *C. quitensis*^41^, as algicolous fungi of macroalgae^47,48^, in freshwater lakes^31^ and in the lichenosphere^49^. Taxonomic studies of *Pseudogymnoascus* draw attention to *P. destructans*, causative agent of the lethal disease white-nose syndrome (WNS) in bats of temperate regions^50^. Further studies are required to elucidate if genetic material of this genus detected here belongs to the *P. destructans* group.

The genus *Mortierella* (*Mortierellomycota*), whose members are also known as “snow moulds”, includes some species often reported in Antarctica. Species of this genus have been reported in association with mosses^39,40^, lichens^49^, soils^24,51^, freshwater^31^, macroalgae^52^ and in the rhizosphere of *Deschampsia antarctica* Desv. (Poaceae)^23^.

Considering specifically the rare taxa detected in the Deception Island fungal assemblages, the sequence data of several taxa detected from Whalers Bay matched fungi previously reported as opportunistically associated with humans and animals or able to cause plant diseases. Amongst these, *M. dermatis*, *R. mucilaginosa* and *T. asahii* (human and animal opportunistic) and *C. lunata*, *G. intricans*, *G. zeae* and *H. juniper* (phytopathogenic) were present in both sampling areas. Although present at apparently low frequency, these fungi merit further attention. For example, de Menezes et al.^33^ reported a high density of cultivable *R. mucilaginosa* in Antarctic snow, a fungus capable of growing at 37 °C and that displays resistance against the antifungal compound fluconazole, and which may represent a health risk for immunosuppressed persons. In this context, Whalers Bay is a very popular visitor site, including by many elderly tourists likely with weaker immune systems, who may therefore come into contact with the resident microorganisms including those reported as opportunistic disease agents. However, further studies are necessary to assess the risk of infection from resident fungi during a visit to Whalers Bay.

The high-throughput sequencing methodology used in the current study allowed detection of the DNA of a range of fungal taxa able to form the lichenized fungal associations, but without their thalli being visibly present in the soils sampled. Although the lichen diversity of mainland Antarctica and adjacent islands is generally well-known^53^, that of Deception Island specifically is less well studied, with 70 species currently reported^53^. Among the species whose fungal DNA was detected in the current study, *V. alpicola*, *T. smaragdula*, *Parmelina* sp., *V. nodosa*, *V. humida* and *V. margacea* are first records for both Deception Island and Antarctica generally. The dominant DNA detected in both sampled areas was that of *V. alpicola*. According to Shivarov et al.^54^ this species is known only from Europe (Austria, Great Britain, Germany, Italy, Norway, Romania, Switzerland). *Trimmatothelopsis smaragdula* is a circumpolar sub-Arctic and alpine species^55^. *Verrucaria humida* is another European lichen known from Wales, Norway, Germany and Poland, while *V. margacea* is widespread in Scandinavia, central and western European mountain ranges, and temperate areas in the Southern Hemisphere^56^ and *V. nodosa* is known only from Wales^57^. Lichens in the genera *Psoroma*, *Lecidea* and *Placopsis* are common in Deception Island and the South Shetland Islands generally.

## Conclusions

DNA metabarcoding of soil fungal assemblages in samples obtained from ASPA 140 subsite B and Whalers Bay on Deception Island indicated the presence of a rich fungal diversity. The ‘rare’ fungal taxa detected in both areas included fungi reported as human and animal opportunistic and plant pathogens. The diversity detected may have been transported to Deception Island associated with human activities such as the historic whaling industry, research, tourism, through natural transport by birds or in the air column, or represent resident fungi not previously described. Further long-term studies are required to elucidate how biological colonization of the island may be affected by climatic changes and other anthropogenic influences.

## Supplementary Information

Supplementary Information.
