# Supplementary material for: DNA metabarcoding uncovers fungal diversity in soils of protected and non-protected areas on Deception Island, Antarctica
Source: Sci Rep. 2020 Dec 15;10:21986. doi: 10.1038/s41598-020-78934-7 (PMC7738542; doi:10.1038/s41598-020-78934-7)
Supplement: Supplementary file 1 — Supplementary Information. [file 41598_2020_78934_MOESM1_ESM.docx]

**DNA metabarcoding uncovers fungal diversity in soils of protected and non-protected areas on Deception Island, Antarctica**

Luiz Henrique Rosa^1^*, Thamar Holanda da Silva^1^, Mayara Baptistucci Ogaki^1^, Otávio Henrique Bezerra Pinto^2^, Michael Stech^3^, Peter Convey^4^, Micheline Carvalho-Silva^5^, Carlos Augusto Rosa^1^ & Paulo EAS Câmara^5^

^1^Departamento de Microbiologia, Universidade Federal de Minas Gerais, Brasil

^2^Departamento de Biologia Celular, Universidade de Brasília, Brasília, Brasil

^3^Naturalis Biodiversity Center, Leiden, Netherlands

^4^British Antarctic Survey, NERC, High Cross, Madingley Road, Cambridge CB3 0ET, United Kingdom

^5^Departamento de Botânica, Universidade de Brasília, Brasília, Brasil

*Corresponding author

Laboratório de Microbiologia Polar e Conexões Tropicais, Departamento de Microbiologia, Instituto de Ciências Biológicas, Universidade Federal de Minas Gerais, Belo Horizonte, MG, P. O. Box 486, CEP 31270-901. Tel.: +55-31-3409 2749; Fax: +55-31-3409 2730, Brazil. E-mail: lhrosa@icb.ufmg.br

**Supplementary Table 1**. Numbers of sequence reads of fungal amplicon sequence variants (ASVs) detected in soil samples at Antarctic Specially Protected Areas (ASPA) 140, subsite B and Whalers Bay on Deception Island.

|  |  | **Sites sampled** | |  |
| --- | --- | --- | --- | --- |
| **Hierarchical level** | **Fungal taxa** | **ASPA 140*** | **Whalers Bay** | **Total** |
| **Fungi** | Fungi sp. | 262844** | 177309 | 440153 |
| **Ascomycota** | *Cladosporium* sp. | 65686 | 30445 | 96131 |
|  | *Pseudogymnoascus roseus* | 62100 | 27730 | 89830 |
|  | *Leotiomycetes* sp. 2 | 1586 | 69884 | 71470 |
|  | *Penicillium* sp. | 12650 | 49477 | 62127 |
|  | *Pseudogymnoascus appendiculatus* | 23236 | 17062 | 40298 |
|  | *Pseudogymnoascus* sp. | 20584 | 13650 | 34234 |
|  | *Cleistothelebolus nipigonensis* | 980 | 12637 | 13617 |
|  | *Thelebolus globosus* | 5326 | 7319 | 12645 |
|  | *Ascomycota* sp. 1 | 4642 | 4461 | 9103 |
|  | *Colletotrichum* sp. 1 | 1093 | 6722 | 7815 |
|  | *Neoascochyta paspali* | 2576 | 3235 | 5811 |
|  | *Chalara pseudoaffinis* | 3705 | 2047 | 5752 |
|  | *Pseudeurotium* sp. | 1764 | 3455 | 5219 |
|  | *Chaetothyriales* sp. 2 | 3656 | 1091 | 4747 |
|  | *Lecanorales* sp. | 2990 | 1141 | 4131 |
|  | *Verrucaria alpicola* | 2305 | 985 | 3290 |
|  | *Diaporthales* sp. | 255 | 2870 | 3125 |
|  | *Helotiales* sp. 1 | 1493 | 1504 | 2997 |
|  | *Helotiales* sp. 2 | 2231 | 718 | 2949 |
|  | *Pseudogymnoascus* sp. | 2004 | 939 | 2943 |
|  | *Dermateaceae* sp. | 116 | 2587 | 2703 |
|  | *Chrysosporium vallenarense* | 1888 | 778 | 2666 |
|  | *Onygenales* sp. 1 | 140 | 2515 | 2655 |
|  | *Didymellaceae* sp. | 21 | 2590 | 2611 |
|  | *Colletotrichum cliviae* | 0 | 2524 | 2524 |
|  | *Penicillium polonicum* | 1199 | 1281 | 2480 |
|  | *Chaetothyriales* sp. 1 | 1590 | 494 | 2084 |
|  | *Chaetomium* sp. | 226 | 1774 | 2000 |
|  | *Verrucariaceae* sp. | 1648 | 294 | 1942 |
|  | *Rhizoscyphus* sp. | 562 | 1216 | 1778 |
|  | *Herpotrichia juniperi* | 598 | 1074 | 1672 |
|  | *Verrucariales* sp. | 1173 | 414 | 1587 |
|  | *Verrucaria* sp. | 1200 | 349 | 1549 |
|  | *Candida boidinii* | 1381 | 147 | 1528 |
|  | *Aspergillus* sp. | 358 | 1142 | 1500 |
|  | *Trichoderma* sp. | 436 | 689 | 1125 |
|  | *Eurotiomycetes* sp. | 1014 | 36 | 1050 |
|  | *Phaeosphaeria dennisiana* | 224 | 727 | 951 |
|  | *Colletotrichum annellatum* | 802 | 127 | 929 |
|  | *Debaryomyces* sp. | 302 | 514 | 816 |
|  | *Psoroma tenue* | 590 | 205 | 795 |
|  | *Penicillium steckii* | 760 | 19 | 779 |
|  | *Pleosporales* sp. 2 | 262 | 420 | 682 |
|  | *Alternaria* sp. | 0 | 667 | 667 |
|  | *Aspergillus sydowii* | 427 | 112 | 539 |
|  | *Capnodiales* sp. | 153 | 381 | 534 |
|  | *Tetracladium marchalianum* | 232 | 294 | 526 |
|  | *Dothideomycetes* sp. | 9 | 503 | 512 |
|  | *Pseudeurotiaceae* sp. | 182 | 306 | 488 |
|  | *Fusarium* sp. | 65 | 359 | 424 |
|  | *Herpotrichiellaceae* sp. | 417 | 0 | 417 |
|  | rthoniales sp. | 260 | 157 | 417 |
|  | *Simplicillium* sp. | 410 | 6 | 416 |
|  | *Cladosporium halotolerans* | 77 | 335 | 412 |
|  | *Penicillium sumatraense* | 359 | 36 | 395 |
|  | *Saccharomycetales* sp. | 351 | 39 | 390 |
|  | *Hypocreales* sp. | 188 | 200 | 388 |
|  | *Leotiomycetes* sp. 1 | 101 | 254 | 355 |
|  | *Oidiodendron truncatum* | 100 | 255 | 355 |
|  | *Penicillium paxilli* | 330 | 0 | 330 |
|  | *Neofusicoccum parvum* | 0 | 318 | 318 |
|  | *Aspergillus niger* | 292 | 18 | 310 |
|  | *Gibberella zeae* | 32 | 278 | 310 |
|  | *Nakazawaea* sp. | 0 | 302 | 302 |
|  | *Penicillium aurantiogriseum* | 55 | 239 | 294 |
|  | *Cylindrocarpon* sp. | 222 | 60 | 282 |
|  | *Aspergillus restrictus* | 81 | 192 | 273 |
|  | *Verrucaria nodosa* | 253 | 0 | 253 |
|  | *Trimmatothelopsis smaragdula* | 73 | 177 | 250 |
|  | *Pseudeurotium hygrophilum* | 233 | 0 | 233 |
|  | *Nectriaceae* sp. | 90 | 141 | 231 |
|  | *Scheffersomyces* sp. | 134 | 97 | 231 |
|  | *Sporormiaceae* sp. | 29 | 198 | 227 |
|  | *Saccharomyces cerevisiae* | 200 | 26 | 226 |
|  | *Mycoleptodiscus* sp. | 0 | 221 | 221 |
|  | *Leptosphaeria sclerotioides* | 87 | 129 | 216 |
|  | *Candida insectorum* | 203 | 0 | 203 |
|  | *Letendraea helminthicola* | 0 | 201 | 201 |
|  | *Chrysosporium* sp. | 0 | 197 | 197 |
|  | *Neodevriesia* sp. | 54 | 143 | 197 |
|  | *Penicillium cairnsense* | 0 | 191 | 191 |
|  | *Cyphellophora* sp. | 74 | 116 | 190 |
|  | *Candida* sp. | 186 | 0 | 186 |
|  | *Xylaria apiculata* | 87 | 98 | 185 |
|  | *Infundichalara* sp. | 131 | 49 | 180 |
|  | *Hyphodiscus* sp. | 38 | 138 | 176 |
|  | *Ascomycota* sp. 2 | 68 | 102 | 170 |
|  | *Microdochium lycopodinum* | 167 | 0 | 167 |
|  | *Pleosporales* sp. 1 | 152 | 13 | 165 |
|  | *Scheffersomyces amazonensis* | 162 | 0 | 162 |
|  | *Sugiyamaella boreocaroliniensis* | 144 | 17 | 161 |
|  | *Aspergillus terreus* | 160 | 0 | 160 |
|  | *Paraconiothyrium* sp. | 45 | 108 | 153 |
|  | *Lecidea cancriformis* | 129 | 21 | 150 |
|  | *Aspergillus penicillioides* | 47 | 102 | 149 |
|  | *Talaromyces sayulitensis* | 0 | 149 | 149 |
|  | *Xylaria cubensis* | 0 | 143 | 143 |
|  | *Candida elateridarum* | 142 | 0 | 142 |
|  | *Fusarium oxysporum* | 0 | 139 | 139 |
|  | *Pezizomycetes* sp. | 0 | 136 | 136 |
|  | *Aureobasidium pullulans* | 46 | 85 | 131 |
|  | *Sporothrix* sp. | 126 | 0 | 126 |
|  | *Chaetosphaeriaceae* sp. | 121 | 0 | 121 |
|  | *Curvularia lunata* | 91 | 29 | 120 |
|  | *Arthrinium hydei* | 0 | 118 | 118 |
|  | *Neopestalotiopsis* sp. | 0 | 118 | 118 |
|  | *Fusarium solani* | 0 | 115 | 115 |
|  | *Onygenales* sp. 2 | 0 | 112 | 112 |
|  | *Cladophialophora* sp. | 109 | 0 | 109 |
|  | *Cladophialophora humicola* | 107 | 0 | 107 |
|  | *Saccharomyces* sp. | 0 | 106 | 106 |
|  | *Phaeosphaeria podocarpi* | 0 | 105 | 105 |
|  | *Kotlabaea* sp. | 0 | 103 | 103 |
|  | *Penicillium brasilianum* | 30 | 68 | 98 |
|  | *Capnodiales* sp. | 94 | 3 | 97 |
|  | *Tetracladium furcatum* | 0 | 97 | 97 |
|  | *Xylaria* sp. 1 | 0 | 90 | 90 |
|  | *Phaeosphaeriaceae* sp. | 89 | 0 | 89 |
|  | *Penicillium citrinum* | 55 | 33 | 88 |
|  | *Penicillium coffeae* | 87 | 0 | 87 |
|  | *Cladosporium sphaerospermum* | 50 | 35 | 85 |
|  | *Pichia membranifaciens* | 58 | 26 | 84 |
|  | *Cladosporium delicatulum* | 58 | 19 | 77 |
|  | *Acremonium charticola* | 52 | 24 | 76 |
|  | *Curvularia* sp. | 0 | 76 | 76 |
|  | *Meyerozyma* sp. | 0 | 76 | 76 |
|  | *Meyerozyma guilliermondii* | 28 | 47 | 75 |
|  | *Candida palmioleophila* | 51 | 22 | 73 |
|  | *Meyerozyma caribbica* | 72 | 0 | 72 |
|  | *Lecanicillium* sp. 1 | 17 | 53 | 70 |
|  | *Talaromyces wortmannii* | 69 | 0 | 69 |
|  | *Tetracladium* sp. | 0 | 68 | 68 |
|  | *Hormonema* sp. | 65 | 0 | 65 |
|  | *Lodderomyces elongisporus* | 29 | 36 | 65 |
|  | *Humicola grisea* | 64 | 0 | 64 |
|  | *Parmelina* sp. | 64 | 0 | 64 |
|  | *Pichia terricola* | 8 | 56 | 64 |
|  | *Colletotrichum* sp. 2 | 39 | 23 | 62 |
|  | *Colletotrichum gigasporum* | 0 | 62 | 62 |
|  | *Candida parapsilosis* | 0 | 60 | 60 |
|  | *Gyoerffyella entomobryoides* | 60 | 0 | 60 |
|  | *Wickerhamomyces anomalus* | 0 | 60 | 60 |
|  | *Microascaceae* sp. | 54 | 0 | 54 |
|  | *Mycosphaerella tassiana* | 0 | 54 | 54 |
|  | *Cyberlindnera* sp. | 34 | 18 | 52 |
|  | *Preussia* sp. | 51 | 0 | 51 |
|  | *Schwanniomyces polymorphus* | 49 | 0 | 49 |
|  | *Penicillium astrolabium* | 48 | 0 | 48 |
|  | *Verrucaria margacea* | 17 | 30 | 47 |
|  | *Gibberella tricincta* | 4 | 42 | 46 |
|  | *Sporothrix brasiliensis* | 0 | 46 | 46 |
|  | *Candida tammaniensis* | 0 | 44 | 44 |
|  | *Diaporthe* sp. | 37 | 7 | 44 |
|  | *Exophiala cancerae* | 43 | 0 | 43 |
|  | *Fusarium asiaticum* | 0 | 43 | 43 |
|  | *Aspergillus flavus* | 42 | 0 | 42 |
|  | *Botryosphaeriaceae* sp. | 28 | 14 | 42 |
|  | *Botryosphaeriales* sp. | 0 | 42 | 42 |
|  | *Aspergillus thermomutatus* | 39 | 0 | 39 |
|  | *Chaetomiaceae* sp. 1 | 21 | 18 | 39 |
|  | *Eurotiales* sp. | 39 | 0 | 39 |
|  | *Arachnomyces* sp. | 0 | 38 | 38 |
|  | *Placopsis* sp. | 35 | 0 | 35 |
|  | *Antennariella placitae* | 34 | 0 | 34 |
|  | *Microdochium tainanense* | 0 | 34 | 34 |
|  | *Microscypha* sp. | 33 | 0 | 33 |
|  | *Fusarium nematophilum* | 11 | 21 | 32 |
|  | *Volucrispora graminea* | 0 | 32 | 32 |
|  | *Oidiodendron setiferum* | 0 | 30 | 30 |
|  | *Penicillium brocae* | 30 | 0 | 30 |
|  | *Sugiyamaella americana* | 0 | 29 | 29 |
|  | *Cosmospora* sp. | 0 | 27 | 27 |
|  | *Starmerella bombicola* | 27 | 0 | 27 |
|  | *Cystodendron* sp. | 26 | 0 | 26 |
|  | *Hamigera* sp. | 0 | 26 | 26 |
|  | *Purpureocillium lavendulum* | 26 | 0 | 26 |
|  | *Volutella consors* | 26 | 0 | 26 |
|  | *Xylaria* sp. 2 | 0 | 26 | 26 |
|  | *Cyphellophora pluriseptata* | 24 | 0 | 24 |
|  | *Psoroma hypnorum* | 24 | 0 | 24 |
|  | *Scheffersomyces coipomensis* | 0 | 24 | 24 |
|  | *Verrucaria humida* | 0 | 24 | 24 |
|  | *Gibberella* sp. | 0 | 23 | 23 |
|  | *Xylariales* sp. | 0 | 22 | 22 |
|  | *Chaetothyriaceae* sp. | 0 | 21 | 21 |
|  | *Pseudallescheria boydii* | 21 | 0 | 21 |
|  | *Barnettozyma californica* | 20 | 0 | 20 |
|  | *Lecanicillium muscarium* | 20 | 0 | 20 |
|  | *Lophiostoma* sp. | 0 | 20 | 20 |
|  | *Aphanoascus keratinophilus* | 0 | 19 | 19 |
|  | *Lecidea* sp. | 0 | 19 | 19 |
|  | *Penicillium catenatum* | 19 | 0 | 19 |
|  | *Penicillium pimiteouiense* | 0 | 19 | 19 |
|  | *Helotiaceae* sp. 2 | 0 | 18 | 18 |
|  | *Peroneutypa scoparia* | 18 | 0 | 18 |
|  | *Phomopsis* sp. | 0 | 18 | 18 |
|  | *Candida heveicola* | 0 | 17 | 17 |
|  | *Coniosporium* sp. | 0 | 17 | 17 |
|  | *Exophiala* sp. | 0 | 17 | 17 |
|  | *Colletotrichum brevisporum* | 0 | 16 | 16 |
|  | *Diatrypaceae* sp. 2 | 16 | 0 | 16 |
|  | *Xylaria hypoxylon* | 0 | 16 | 16 |
|  | *Gliomastix tumulicola* | 0 | 15 | 15 |
|  | *Gibberella intricans* | 14 | 0 | 14 |
|  | *Chaetomiaceae* sp. 2 | 13 | 0 | 13 |
|  | *Hanseniaspora lachancei* | 0 | 13 | 13 |
|  | *Helotiaceae* sp. 1 | 0 | 13 | 13 |
|  | *Pichia kluyveri* | 13 | 0 | 13 |
|  | *Sordariomycetes* sp. | 0 | 13 | 13 |
|  | *Blastobotrys proliferans* | 0 | 12 | 12 |
|  | *Hanseniaspora* sp. | 4 | 8 | 12 |
|  | *Pestalotiopsis trachicarpicola* | 0 | 12 | 12 |
|  | *Talaromyces* sp. | 0 | 12 | 12 |
|  | *Purpureocillium* sp. | 0 | 11 | 11 |
|  | *Pyronemataceae* sp. | 0 | 10 | 10 |
|  | *Pyrenochaeta keratinophila* | 0 | 8 | 8 |
|  | *Talaromyces atroroseus* | 0 | 8 | 8 |
|  | *Tolypocladium album* | 0 | 8 | 8 |
|  | *Aspergillus gracilis* | 0 | 7 | 7 |
|  | *Diatrypaceae* sp. 1 | 7 | 0 | 7 |
|  | *Nigrospora oryzae* | 2 | 5 | 7 |
|  | *Cadophora malorum* | 0 | 6 | 6 |
|  | *Leotiomycetes* sp. 3 | 0 | 6 | 6 |
|  | *Periconia byssoides* | 0 | 6 | 6 |
|  | *Byssochlamys spectabilis* | 0 | 5 | 5 |
|  | *Muscodor fengyangensis* | 0 | 5 | 5 |
|  | *Magnusiomyces capitatus* | 0 | 4 | 4 |
|  | *Lecanicillium* sp. 2 | 0 | 3 | 3 |
|  | *Pezizaceae* sp. | 2 | 0 | 2 |
| ***Basidomycota*** | *Leucosporidiella creatinivora* | 4748 | 18853 | 23601 |
|  | *Malassezia restricta* | 11413 | 3719 | 15132 |
|  | *Apiotrichum dehoogii* | 1910 | 4890 | 6800 |
|  | *Malassezia globosa* | 5831 | 689 | 6520 |
|  | *Rhodotorula mucilaginosa* | 2565 | 2663 | 5228 |
|  | *Malassezia* sp. 2 | 2643 | 400 | 3043 |
|  | *Agaricomycetes* sp. 2 | 1292 | 1109 | 2401 |
|  | *Mrakia frigida* | 712 | 1182 | 1894 |
|  | *Rhodotorula ingeniosa* | 1443 | 95 | 1538 |
|  | *Mycena* sp. | 192 | 1287 | 1479 |
|  | *Pucciniomycetes* sp. | 286 | 1154 | 1440 |
|  | *Malasseziales* sp. | 1112 | 321 | 1433 |
|  | *Leucosporidiaceae* sp. | 301 | 1060 | 1361 |
|  | *Basidiomycota* sp. 1 | 927 | 411 | 1338 |
|  | *Vishniacozyma victoriae* | 298 | 1031 | 1329 |
|  | *Rhodotorula* sp. 2 | 780 | 390 | 1170 |
|  | *Leucosporidiales* sp. | 646 | 439 | 1085 |
|  | *Microbotryomycetes* sp. 2 | 597 | 445 | 1042 |
|  | *Marasmius* sp. | 602 | 0 | 602 |
|  | *Trichosporon* sp. | 219 | 351 | 570 |
|  | *Malassezia* sp. 1 | 439 | 104 | 543 |
|  | *Malassezia sympodialis* | 364 | 171 | 535 |
|  | *Malassezia equina* | 415 | 0 | 415 |
|  | *Papiliotrema laurentii* | 0 | 358 | 358 |
|  | *Tremellomycetes* sp. | 59 | 230 | 289 |
|  | *Glaciozyma martinii* | 155 | 117 | 272 |
|  | *Dioszegia changbaiensis* | 133 | 107 | 240 |
|  | *Trichosporon asahii* | 158 | 68 | 226 |
|  | *Agaricales* sp. | 158 | 64 | 222 |
|  | *Ustilaginaceae* sp. | 220 | 0 | 220 |
|  | *Thanatephorus cucumeris* | 23 | 182 | 205 |
|  | *Cortinarius* sp. | 187 | 0 | 187 |
|  | *Perenniporia* sp. | 35 | 83 | 118 |
|  | *Trechisporales* sp. 1 | 0 | 118 | 118 |
|  | *Ceriporia* sp. | 116 | 0 | 116 |
|  | *Psathyrella rostellata* | 114 | 0 | 114 |
|  | *Coprinellus disseminatus* | 0 | 108 | 108 |
|  | *Malassezia dermatis* | 36 | 70 | 106 |
|  | *Peniophora laxitexta* | 99 | 0 | 99 |
|  | *Pseudozyma jejuensis* | 96 | 0 | 96 |
|  | *Vishniacozyma dimennae* | 0 | 83 | 83 |
|  | *Sporisorium* sp. | 81 | 0 | 81 |
|  | *Ceratobasidiaceae* sp. | 0 | 73 | 73 |
|  | *Hypholoma frowardii* | 68 | 0 | 68 |
|  | *Phellinus gilvus* | 68 | 0 | 68 |
|  | *Malassezia yamatoensis* | 0 | 65 | 65 |
|  | *Cutaneotrichosporon smithiae* | 0 | 64 | 64 |
|  | *Psathyrella* sp. | 63 | 0 | 63 |
|  | *Agaricomycetes* sp. 1 | 46 | 16 | 62 |
|  | *Coprinellus xanthothrix* | 62 | 0 | 62 |
|  | *Byssomerulius corium* | 0 | 59 | 59 |
|  | *Apiotrichum* sp. | 58 | 0 | 58 |
|  | *Corticiales* sp. 2 | 58 | 0 | 58 |
|  | *Leucosporidium* sp. | 23 | 34 | 57 |
|  | *Tremella fuciformis* | 50 | 6 | 56 |
|  | *Cantharellales* sp. | 0 | 55 | 55 |
|  | *Apiotrichum xylopini* | 54 | 0 | 54 |
|  | *Irpex hydnoides* | 49 | 0 | 49 |
|  | *Gymnopus* sp. 1 | 44 | 0 | 44 |
|  | *Hannaella* *phetchabunensis* | 0 | 38 | 38 |
|  | *Goffeauzyma* sp. | 30 | 7 | 37 |
|  | *Malassezia slooffiae* | 35 | 0 | 35 |
|  | *Stereum* sp. | 34 | 0 | 34 |
|  | *Saitozyma podzolica* | 0 | 32 | 32 |
|  | *Basidiomycota* sp. 2 | 31 | 0 | 31 |
|  | *Trichosporonaceae* sp. | 0 | 31 | 31 |
|  | *Corticium* sp. | 30 | 0 | 30 |
|  | *Coprinellus* sp. | 29 | 0 | 29 |
|  | *Ganodermataceae* sp. | 0 | 28 | 28 |
|  | *Corticiales* sp. 1 | 27 | 0 | 27 |
|  | *Punctularia strigosozonata* | 25 | 0 | 25 |
|  | *Schizophyllum commune* | 25 | 0 | 25 |
|  | *Filobasidium magnum* | 0 | 24 | 24 |
|  | *Pholiota* s sp. | 0 | 23 | 23 |
|  | *Heterochaete shearii* | 0 | 22 | 22 |
|  | *Fistulina hepatica* | 19 | 0 | 19 |
|  | *Austropaxillus squarrosus* | 18 | 0 | 18 |
|  | *Auriculariales* sp. | 16 | 0 | 16 |
|  | *Saitozyma flava* | 0 | 10 | 10 |
|  | *Gymnopus* sp. 2 | 0 | 8 | 8 |
|  | *Trechisporales* sp. 2 | 0 | 8 | 8 |
|  | *Postia dissecta* | 0 | 7 | 7 |
|  | *Microbotryomycetes* sp. 1 | 0 | 5 | 5 |
|  | *Peniophora albobadia* | 5 | 0 | 5 |
|  | *Ustilago* sp. | 4 | 0 | 4 |
|  | *Rhodotorula* sp. 1 | 0 | 3 | 3 |
|  | *Trechispora* sp. | 0 | 2 | 2 |
| ***Chytridiomycota*** | *Chytridiomycota* sp. 2 | 2578 | 5802 | 8380 |
|  | *Powellomyces* sp. | 1771 | 0 | 1771 |
|  | *Protrudomyces lateralis* | 515 | 272 | 787 |
|  | *Chytridiomycota* sp. 1 | 175 | 159 | 334 |
|  | *Paludomyces mangrovei* |  | 77 | 77 |
|  | *Lobulomycetaceae* sp. | 5 | 11 | 16 |
|  | *Lobulomyces angularis* | 6 | 0 | 6 |
|  | *Clydaea vesicula* | 4 | 0 | 4 |
| ***Mortierellomycota*** | *Mortierella* sp. 1 | 9035 | 45562 | 54597 |
|  | *Mortierella* sp. 2 | 12802 | 33797 | 46599 |
|  | *Mortierella fimbricystis* | 8842 | 4015 | 12857 |
|  | *Mortierella gamsii* | 2443 | 5923 | 8366 |
|  | *Mortierella antarctica* | 6589 | 642 | 7231 |
|  | *Mortierella alpina* | 925 | 1014 | 1939 |
|  | *Mortierella elongatula* | 615 | 29 | 644 |
|  | *Mortierella globalpina* | 459 | 85 | 544 |
|  | *Mortierella parvispora* | 212 | 208 | 420 |
|  | *Mortierella sclerotiella* | 164 | 63 | 227 |
|  | *Mortierella exigua* | 85 | 0 | 85 |
|  | *Mortierella turficola* |  | 78 | 78 |
|  | *Mortierella hyalina* | 0 | 11 | 11 |
|  | *Mortierella minutissima* | 5 | 0 | 5 |
| ***Mucoromycota*** | *Densospora* sp. | 2798 | 498 | 3296 |
|  | *Syncephalastrum monosporum* | 19 | 0 | 19 |
|  | *Mucoromycota* sp. | 11 | 0 | 11 |
|  | *Gongronella* sp. | 0 | 9 | 9 |
| ***Rozellomycota*** | *Rozellomycota* sp. 1 | 932 | 1899 | 2831 |
|  | *Rozellomycota* sp. 2 | 0 | 40 | 40 |

* ASPA = Antarctic Specially Protected Area; ** = number of the reads.
